# Smartphone-Based Ecological Momentary Assessment of Coping with Loneliness amid COVID-19 in Germany

**DOI:** 10.3390/ijerph19073946

**Published:** 2022-03-26

**Authors:** Luisa Wegner, Matthias N. Haucke, Stephan Heinzel, Shuyan Liu

**Affiliations:** 1 Department of Psychiatry and Psychotherapy, Campus Charité Mitte, Charité—Universitätsmedizin Berlin, 10117 Berlin, Germany; matthias.haucke@charite.de or; 2 Clinical Psychology and Psychotherapy, Department of Education and Psychology, Freie Universität Berlin, 14195 Berlin, Germany; stephan.heinzel@fu-berlin.de

**Keywords:** perceived social isolation, problem-focused coping, emotion-focused coping, relationship-focused coping, second national lockdown

## Abstract

The COVID-19 pandemic may have caused people to feel isolated, left out, and in need of companionship. Effective strategies to cope with such unrelenting feelings of loneliness are needed. In times of COVID-19, we conducted a smartphone-based ecological momentary assessment (EMA) study with 280 lonely participants in Germany over 7 months, where a long and hard second national lockdown was in place. Each participant reported their daily loneliness and coping strategies for loneliness once in the evening for 7 consecutive days. We found that managing emotions and social relationships were associated with decreased feelings of loneliness, while using a problem-focused coping strategy was associated with increased feelings of loneliness amid COVID-19. Interestingly, managing emotions was particularly effective for easing loneliness during the second lockdown. Females tend to use more emotion-focused coping strategies to overcome their loneliness compared to males. Our study highlights the importance of managing emotions against loneliness throughout the COVID-19 pandemic in Germany. Designing technology that provides emotional support to people may be one of the keys to easing loneliness and promoting well-being.

## 1. Introduction

Loneliness is the experience of a discrepancy between one’s desired and actual social connection [1,2]. It has a substantial adverse impact on mental and physical health [3], as well as serious consequences for social cohesion, trust and participation [4]. Social restrictions adopted to prevent the spread of COVID-19 have had unintended side effects, such as experiencing loneliness and social isolation [5,6]. Thus, greater attention to effective coping strategies is needed.

A survey by the European Commission shows that due to the COVID-19 pandemic, feelings of loneliness among EU residents in Europe doubled from 12% in 2016 to 25% in spring 2020 [5]. The latest report reveals that over one in three people in the United States faced “serious loneliness” during the pandemic [7]. Besides a substantial increase in prevalence, loneliness persisted even without a lockdown [8]. After the first lockdown, 66% of respondents in Germany reported feeling loneliness between summer and early autumn 2020 [8] compared to 32% reporting to be lonely in May 2020 [9]. Moreover, after the lifting of a strict lockdown, loneliness not only outlasted but also increased negative mood including fatigue, anxiety, stress, depression and unhappiness [10]. The long-term impact of loneliness on mental health can no longer be ignored as well: Spanning the period from May 2020 to March 2021, the prevalence of loneliness in Germany was found to be associated with increased long-term psychological distress [11]. While increasing concerns about a “loneliness epidemic” and its relation to wellbeing is gaining momentum [12,13], effective coping strategies for loneliness are still largely neglected [14].

The tripartite model of coping strategies suggests that coping involves three factors: problem-, emotion- and relationship-focused coping [15]. Problem-focused coping describes efforts to resolve the problem (e.g., trying to solve one’s problems), emotion-focused coping involves trying to reduce the negative emotional responses (e.g., accepting one’s feelings and focusing on what really matters), and relationship-focused coping refers to efforts to manage social relationships (e.g., turning to friends for companionship and support) [15]. Before COVID-19, a review showed that problem-focused coping strategies were associated with lower levels of loneliness, and emotion-focused coping with higher levels of loneliness, indicating problem-focused coping is a key aspect in designing interventions targeting loneliness [16]. However, when individuals could not change a situation, they directed their efforts from problem-focused coping to emotion-focused coping [15].

In times of the COVID-19 pandemic, countries in lockdowns have seen an exponential rise in feelings of loneliness as limited opportunities for in-person social interactions existed [17]. Coping behaviors and social support were identified as protective factors against loneliness [18]. Under the first lockdown, positive emotion-focused coping styles (e.g., acceptance) were associated with better mental health [19]. Sharing thoughts and feelings about COVID-19 with others also reduced feelings of loneliness [20]. Moreover, lonely individuals were inclined to use social media to cope with lacking social contact [21]. In addition to different coping strategies, recent studies suggested that there are gender differences in adopting coping strategies [22,23]. Females opted for emotion-focused coping in response to COVID-19 more than males [23].

While most studies were carried out during the first national lockdown [18,20,21,24], there is a shortage of longitudinal measurement in the prolonged pandemic, especially throughout the second COVID-19 lockdown in Germany which was unexpectedly longer and harder than the first. Most previous studies conducted by online surveys [18,20,21] may lack ecological validity that allows for the momentary assessment of loneliness and its coping strategies in everyday life. It is also unclear if all three factors of the tripartite model are effective strategies for coping with loneliness over the course of COVID-19 and if a longer and harder second lockdown brought a significant change in effective coping strategies in comparison to pre-second lockdown. That is of particular relevance in the light of the current response to loneliness and the future preparedness for a potential world loneliness crisis.

We conducted an ecological momentary assessment (EMA) in Germany. We investigated strategies that lonely individuals use to cope with loneliness amid COVID-19 and specifically compared the course of COVID-19 during the pre-second (8 August 2020–1 November 2020) versus the second (2 November 2020–9 March 2021) lockdown. The second lockdown included the following measures: (1) maximally five people from two households were allowed to meet indoors, and maximally 10 people from two households were allowed to meet outdoors; (2) institutions and cultural, sport and leisure facilities were closed; (3) retail shops that were not necessary for daily life were closed; (4) restaurants were closed and were only available for take-out or delivery; (5) travel restrictions abroad and within country; (6) hotels were forbidden to host guests for vacation purposes [25]. Given the evidence of an increase in loneliness during the first lockdown [5,8], we hypothesized that a long and hard second lockdown will further increase the feeling of loneliness. With such an increase in loneliness, lonely individuals will use all three types of coping strategies (i.e., problem-, emotion-, and relationship-focused coping). Emotion-and relationship-focused coping will be more effective than problem-coping under the restricted circumstances that individuals can change [15]. Lastly, we expected that there will be gender differences in adopting coping strategies [22,23].

## 2. Materials and Methods

### 2.1. Participants and Procedure

We enrolled 280 out of 1549 participants for an EMA study between 8 August 2020 and 13 March 2021. Each participant underwent 7 consecutive days of EMA. The enrolled participants were (1) aged 18 years or older, (2) not working a night shift, (3) using an Android smartphone, (4) speaking fluent German, and at least sometimes feeling lonely according to a short 8-item UCLA Loneliness Scale (ULS-8; cut-off score = 16, indicating mild trait loneliness [26] as the scope of this study was to explore coping strategies among this population). A total of 1269 out of 1549 participants were excluded by not meeting inclusion criteria (N = 854) or not willing to participate (N = 415). The detailed items of ULS-8 questionnaires can be found in our previous study [8]. The study was approved by both the Ethics Committee of Charité—Universitätsmedizin Berlin (Registration number: EA2/143/20) and the Ethics Committee of Freie Universität Berlin (Registration number: 030/2020).

### 2.2. Ecological Momentary Assessment

We performed the ecological momentary assessment (EMA) on the smartphone application “movisensXS” (movisens GmbH, Karlsruhe, Germany). The EMA consisted of a socio-demographic assessment (e.g., age, gender, and years of education) and repeated sampling of participants’ real-time real-life behaviors and experiences for 7 consecutive days. To measure the everyday experience of loneliness and reduce participant fatigue and burden, we used a short 3-item UCLA Loneliness Scale (ULS-3) [27] to ask participants to rate on a visual analogue scale (0–100: 0 = not at all, 100 = extremely) once a day in the evening. It measured how often they felt a lack of companionship, left out, or isolated from others. Daily coping with loneliness was measured once a day in the evening by using the Coping with Loneliness Questionnaire [28], on a visual analogue scale (0–100: 0 = not at all, 100 = extremely). All items started with “Today…”. The items were retrieved from problem-focused coping (“I decided to face and try to solve my problems”), emotion-focused coping (“I came to accept how I felt” and “I tried to focus on what really mattered to me in life”), and relationship-focused coping (“I turned to my friends for companionship and support”) [15].

### 2.3. Data Analysis

We conducted our statistical analyses in the R version 4.1.0 Statistical Software. To test whether loneliness increased during the second national lockdown, we built up a multiple linear regression model (“COVID-19 lockdown” as the predictor and “daily loneliness” as the outcome) by controlling for age, gender, years of education, and individuals’ trait loneliness scores. To test the effectiveness of problem-, emotion-, and relationship-focused coping strategies on easing loneliness through pre-second and second lockdown, we performed a multilevel hierarchical regression model with the outcome variable “daily loneliness” and three predictors from daily coping with loneliness “problem-focused coping”, “emotion-focused coping”, and “relationship-focused coping” and another predictor “COVID-19 lockdown” (*pre-second lockdown* versus *second lockdown*; effect coding: +0.5 versus −0.5) as fixed effects by controlling for age, gender, years of education, and individuals’ trait loneliness scores. We recomputed the predictors by centering around each person’s mean. To meet the assumption of having no multicollinearity, we calculated the variance inflation factor (VIF) values for all predictors of the model. In addition, we included random effects following a model comparison approach. We compared the models based on log-likelihood ratios; the results were consistent when judged by the Akaike information criterion (AIC) [29] or the Bayesian information criterion (BIC) [30]. To determine the effectiveness of different coping strategies between pre-second and second lockdown, we conducted subgroup analysis for pre-second and second lockdown groups with the best fitted model separately. To assess the gender differences in adopting coping strategies, we conducted subgroup analyses for males and females with the best fitted model separately.

## 3. Results

**Figure 1:**
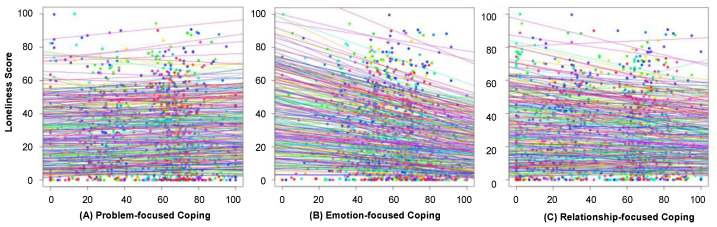
(**A**) Problem-focused coping was significantly associated with increased daily loneliness levels. (**B**) Emotion- and (**C**) relationship-focused coping was significantly associated with decreased daily loneliness levels. Regression lines are based on the random intercept and random slope for each individual participant and are based on seven observations for each participant.

In total, 280 participants in Germany (190 females; age range: 18–72, Mean = 30.96, SD = 11.33) completed our EMA study. We found an increase in loneliness in the second lockdown as compared to pre-second lockdown (b = −3.08, *t*(1747) = 2.68, *p* < 0.001). In general, our 7-month study showed that problem-focused coping (b = 0.07, *t*(264) = 2.95, *p* = 0.003) was significantly associated with increased feeling of loneliness, whereas emotion-focused (b = −0.14, *t*(264) = −4.98, *p* < 0.001) and relationship-focused coping (b = −0.07, *t*(264) = −2.99, *p* = 0.003) were significantly associated with decreased daily loneliness, as shown in Figure 1. There were no multicollinearity issues between the outcome of daily loneliness and all the predictors (all VIF values < 1.09). Moreover, we found that there was a change in coping strategies for loneliness during the second lockdown. Emotion-focused coping was significantly effective for easing loneliness (b = −0.15, *t*(130) = −3.88, *p* < 0.001) and relationship-focused coping had no effect on loneliness (b = −0.06, *t*(130) = −1.76, *p* = 0.081) during the second lockdown, although both emotion- and relationship-focused coping were effective for easing loneliness during the pre-second lockdown (both *p* values < 0.006).

**Figure 2:**
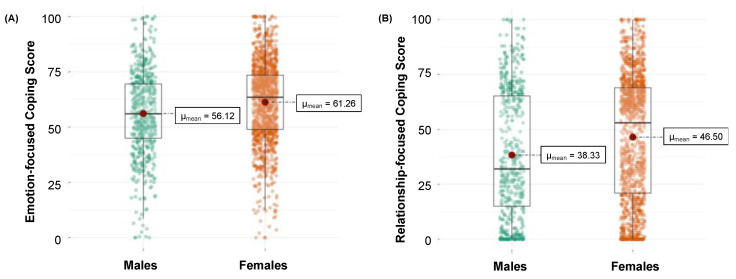
Gender difference in (**A**) emotion- and (**B**) relationship-focused coping.

Regarding gender differences in adopting coping strategies, we found that emotion-focused and relationship-focused coping were significantly associated with a decrease in daily loneliness in both males and females (all *p* values < 0.049). In comparison to males, females used more emotion-focused (*t*(984.72) = −5.29, *p* < 0.001) and relationship-focused (*t*(1036.7) = −5.54, *p* < 0.001) coping styles, as shown in Figure 2. Problem-focused coping was significantly associated with increased feelings of loneliness among males only (b = 0.11, *t*(80) = 2.68, *p* = 0.008) and was not significantly associated with an increase in loneliness among females (b = 0.05, *t*(184) = 1.81, *p* = 0.071).

## 4. Discussion

We conducted an EMA study in Germany over 7 months to investigate if certain coping strategies correlated with decreased feelings of loneliness amid COVID-19 in general, and particularly during a long and hard second lockdown. In line with our hypothesis, we found that trying to focus on what really mattered in one’s life and turning to friends for companionship and support were associated with reducing loneliness amid COVID-19 in general. Together with an increase in loneliness, emotion-focused coping was particularly effective for easing loneliness during the second lockdown. Interestingly, females opted for emotion-focused coping for loneliness more than males. Contrary to our hypothesis, deciding to face and trying to solve problems (problem-focused coping) seemed to stand in relation to increased feelings of loneliness.

Our results may reflect that COVID-19 related cannot be fixed immediately. In situations that cannot be solved or are out of control, such as the loss of a loved one, persistently engaging in problem-focused coping efforts may become maladaptive, in turn, increasing loneliness [15,31]. Instead of problem-focused coping, we found that managing social relationships plays a positive role in reducing loneliness. Lonely individuals often show positive attitudes to build and maintain social bonds [32]. However, a long and hard second lockdown might be less conducive to the cultivation and seeking of social connections, which can contribute to higher feelings of loneliness compared to the pre-second lockdown. This may also be explained by general negative expectations about social relationships among lonely individuals [32]. Lonely individuals are at risk of a circular process with loneliness experiences resulting in considering lowering one’s expectations about relationships, which results in a greater likelihood of loneliness, thus contributing to sustaining or re-establishing loneliness [32]. A reduced sense of being significant and cared for by others could also lead to loneliness, which highlights the importance of forming strong social relationships against feelings of loneliness, particularly with peers who make one feel important and cared for [33].

Our results show that emotion-focused coping was an effective strategy to alleviate higher levels of loneliness during the second lockdown. Females used more emotion-focused coping styles for loneliness than males. These results are consistent with earlier research during the first lockdown suggesting that positive emotion-focused coping was associated with better mental health [19] and that females’ coping styles are highly emotion-focused [23].

Considering that managing emotions had the strongest link to easing loneliness compared to resolving problems and managing social relationships, our study may scale up intervention studies that encourage and support lonely individuals to manage their emotions. Higher emotional stability has also been shown to be associated with lower loneliness [34]. Our results also highlight that future research on loneliness during COVID-19 may include emotional or social components by combining digital interventions. A recent study showed that online social connections mediate the relationship between loneliness and positive coping strategies, indicating that if participants engaged more with others via the internet or different social media platforms, the more likely they were to engage in positive coping strategies to overcome loneliness [35].

Our study did not investigate other types of coping strategies beyond the three types in the tripartite model. People may differ in particular styles of coping or prefer to use certain coping strategies over others. Other effective interventions for loneliness during COVID-19 include psychological therapy (e.g., mindfulness, Tai Chi meditation, and laughter therapy), educational programs (e.g., lessons on friendship and social integration) and social facilitation with peers [14]. Special attention goes to avoiding maladaptive coping behaviors [36]. The generalizability of the results is limited by the absence of participants’ personality characteristics or social skills that may contribute to the ability to alleviate loneliness [15,37]. Adding to that, the possible impact of one’s individual loneliness on coping behaviour and their bidirectional relationship were not explored [16,31]. We examined daily state loneliness but did not assess individuals’ sources of loneliness. Future studies may identify coping strategies tailored to an individual’s source of loneliness as well as how both influence each other. Despite these potential limitations, our study provides the strongest insight yet into potentially effective emotion-focused coping strategies in the face of a “loneliness epidemic” [12,13].

## 5. Conclusions

We conducted an EMA study in Germany to investigate strategies that lonely individuals used to cope with loneliness over the course of the second lockdown. With a rising prevalence of loneliness, we found that lonely individuals used emotion- and relationship-focused coping styles to overcome their feelings of loneliness amid COVID-19. Managing emotions was particularly effective for easing loneliness during the second lockdown. Females exhibited more emotion-focused coping style to overcome their loneliness than males. Our study sheds light on how lonely individuals coped with their loneliness feelings, highlighting the important role that managing emotions plays in coping strategies. In responses to the COVID-19 pandemic, lockdown and a “loneliness epidemic”, future studies may explore the use of digital technology for helping to build and strengthen emotional support networks.

## Data Availability

Derived data supporting the findings of this study are available from the corresponding author, S.L., upon reasonable request.
